# Reproductive issues in carriers of germline pathogenic variants in the *BRCA1/2* genes: an expert meeting

**DOI:** 10.1186/s12916-021-02081-7

**Published:** 2021-09-10

**Authors:** Barbara Buonomo, Claudia Massarotti, Miriam Dellino, Paola Anserini, Alberta Ferrari, Maria Campanella, Mirosa Magnotti, Cristofaro De Stefano, Fedro Alessandro Peccatori, Matteo Lambertini

**Affiliations:** 1 Fertility and Procreation Unit, Gynecologic Oncology Program, European Institute of Oncology IRCCS, Milan, Italy; 2 Department of Neurosciences, Rehabilitation, Ophthalmology, Genetics, Maternal and Child Health (DiNOGMI), School of Medicine, University of Genova, Genova, Italy; 3 Academic Unit of Obstetrics and Gynaecology, IRCCS Ospedale Policlinico San Martino, Genova, Italy; 4 Gynecologic Oncology Unit, IRCCS Istituto Tumori “Giovanni Paolo II”, Bari, Italy; 5 Physiopathology of Human Reproduction Unit, IRCCS Ospedale Policlinico San Martino, Genova, Italy; 6 Department of Surgical Sciences, General Surgery III-Breast Surgery, Fondazione IRCCS Policlinico San Matteo, and Department of Clinical Surgical Sciences, University of Pavia, Pavia, Italy; 7 aBRCAdabra, National Patient Advocacy Association for carriers of BRCA genes mutation, Palermo, Italy; 8 ACTO Campania, Alleanza Contro il Tumore Ovarico, Avellino, Italy; 9 Department of Women’s and Children’s Health, “San Giuseppe Moscati” Hospital, Avellino, Italy; 10 Department of Internal Medicine and Medical Specialties (DiMI), School of Medicine, University of Genova, Genova, Italy; 11 Department of Medical Oncology, UOC Clinica di Oncologia Medica, IRCCS Ospedale Policlinico San Martino, Genova, Italy

**Keywords:** Fertility, *BRCA*, Preimplantation genetic testing, Oocyte cryopreservation, Ovarian reserve, Cancer

## Abstract

**Background:**

Healthy individuals and patients with cancer who are carriers of germline pathogenic variants in the *BRCA1/2* genes face multiple reproductive challenges that require appropriate counseling and specific expertise.

**Main body:**

On December 5th–7th, 2019, patient advocates and physicians with expertise in the field of reproductive medicine, fertility preservation, and oncology were invited to “San Giuseppe Moscati” Hospital in Avellino (Italy) for a workshop on reproductive management of women with germline pathogenic variants in the BRCA1/2 genes. From the discussion regarding the current evidence and future prospective in the field, eight main research questions were formulated and eight recommendations were developed regarding fertility, fertility preservation, preimplantation genetic testing, and pregnancy in healthy carriers and patients with cancer.

**Conclusion:**

Several misconceptions about the topic persist among health care providers and patients often resulting in a discontinuous and suboptimal management. With the aim to offer patient-tailored counseling about reproductive issues, both awareness of current evidences and research should be promoted.

## Background

About 5% to 10% of all cancers are related to germline pathogenic variants in cancer-susceptibility genes [1]. The most frequent predisposing genetic alterations are in the *BRCA1* and *BRCA2* genes, with a cumulative lifetime risk for breast cancer (BC) of 72% and 69% for carriers of pathogenic variants in *BRCA1* and *BRCA2*, respectively [2]. The cumulative ovarian cancer (OC) risk is 44% for *BRCA1* and 17% for *BRCA2* carriers [2]. In the majority of health systems, the access to genetic testing depends on a family-history model [3]; thus, the detection of *BRCA1/2* carriers before a cancer diagnosis remains limited. However, their identification has become more common thanks to several factors such as the therapeutic implications of genetic testing in some diseases (including epithelial OC, BC, pancreatic cancer, and prostate cancer) as well as the development and widespread use of multi-gene panel sequencing technologies [4, 5]. Therefore, there is a frequent need of expert counseling not only for patients with *BRCA*-related cancers, but also for the so-called “previvors” (i.e., individuals that have a *BRCA* pathogenetic variant without being affected by cancer). This counseling includes a discussion on risk-reducing interventions (including risk-reducing salpingo-oophorectomy [RRSO] at the age of 35–40 years for *BRCA1* carriers and between 40 and 45 years for *BRCA2* carriers) which temporarily or permanently impact on lifestyle, body image, endocrinological status, and reproductive choices [6]. Thus, previvors and *BRCA1/2*-mutated patients with cancer face multiple concerns in terms of reproductive challenges that should be separately addressed [7].

In the present manuscript, we summarize and discuss controversial issues in the field of reproductive medicine in young women carrying germline pathogenic variants in the *BRCA1/2* genes and report recommendations from a multidisciplinary group of experts.

## Main text

On December 5th–7th, 2019, patient advocates and physicians with expertise in the field of reproductive medicine, fertility preservation (FP), and oncology were invited to “San Giuseppe Moscati” Hospital in Avellino (Italy) to participate in a workshop on the reproductive management of women with germline pathogenic variants in the *BRCA1/2* genes. The invited experts represented different disciplines related to the topic including oncologists, gynecologists, geneticists, surgeons, and bioethic specialists. Starting from patients’ needs voiced by the advocates present at the workshop, a total of 8 controversial issues were discussed. Experts were asked to present an up-to-date overview of the preclinical and clinical literature available on these topics. On the basis of the data presented, 8 statements were developed.

### Question 1: Are pregnancy and breastfeeding safe in *BRCA* pathogenic variant carriers?

Several studies investigated the effect of parity on BC risk in healthy *BRCA* pathogenic variant carriers [8]. This topic remains controversial for the reported differences in *BRCA1* and *BRCA2* pathogenic variant carriers and for the different effects of pregnancy on breast cancer risk according to age also in the general non-*BRCA* carrier population. A large prospective study showed that women with *BRCA1* pathogenic variants who had two, three, four, or more full-term pregnancies were at 21%, 30%, and 50% decreased risk of BC compared to women with a single full-term pregnancy [9]. On the contrary, women with *BRCA2* pathogenic variants with multiple pregnancies had a significantly increased risk of developing BC [10]. In the general population, healthy women have a transient increased risk of BC after a pregnancy and the increased risk is higher for women with a family history of BC and for women with a pregnancy at a later age [11, 12]. This increased risk has been attributed to the growth-promoting effect of the endocrinological milieu of pregnancy on existing pre-malignant or malignant breast cancer lesions that occur more frequently at an advanced age [11]. It is possible, even if it has never been proved, that *BRCA2* mutation carriers harbor more pre-malignant estrogen receptor-positive lesions and that repeated pregnancies might increase the risk of developing breast cancer [13].

The same differences between *BRCA1* and *BRCA2* carriers are also apparent regarding the effect of breastfeeding on breast cancer risk. In *BRCA1* pathogenic variant carriers, breastfeeding for at least 1 year reduces BC risk (OR = 0.68; 95% CI 0.52 to 0.91; *P* = 0.008), while no effect has been described for healthy *BRCA2* carriers [14]. Thus, the timing of risk-reducing bilateral mastectomy in healthy *BRCA* carriers remains a matter of discussion. On one hand, removing the breasts before pregnancy consistently reduces the risk of subsequent BC and the need of careful monitoring during pregnancy and breastfeeding. On the other hand, many women highly value the advantages of breastfeeding their infants [15] and are reluctant to undergo surgery before pregnancy. For the above-mentioned considerations, it appears clear that the counseling of young *BRCA* pathogenic variant carriers seeking pregnancy is complex and should consider risk estimations according to family history, age, and breast density, but also the personal values on reproductive choices, that remain extremely sensitive and personal. If the woman chooses to maintain her breasts and postpone risk-reducing bilateral mastectomy, a careful monitoring with breast ultrasound during pregnancy and with breast ultrasound and mammogram during breastfeeding should be planned [16]. To reduce diagnostic delay, it is of utmost importance that women who are *BRCA* pathogenic variant carriers and their physicians are aware of the possibility of breast cancer occurrence also during pregnancy and breastfeeding.

To further complicate the issue of pregnancy and breastfeeding in *BRCA* pathogenic variant carriers, we should consider the available data on the effect of subsequent pregnancy in the population of *BRCA* carriers already affected by BC. In this group of patients, current data show that subsequent pregnancy does not increase breast cancer-related events [17, 18]. A large international study has recently shown that, independently of the receptor status and especially for *BRCA1* pathogenic variants carriers, pregnancy after BC seems to be safe without negative consequences on maternal prognosis or fetal outcomes [19]. Even if this study included a significant number of patients, it had some limitations including short-term follow-up (~ 4 year follow-up since the pregnancy) and limited power to detect differences particularly in *BRCA2* carriers [19]. Moreover, the retrospective nature of this and other studies represents an important limitation that does not allow to derive definitive and strong conclusions [20]. In BC patients that need 5 to 10 years of adjuvant endocrine therapy (ET) including *BRCA* carriers, an international clinical trial, which has recently completed the target accrual, is assessing the safety and feasibility of a temporary interruption of ET after at least 18 months, in order to allow pregnancy [21].

*Recommendation 1: In healthy BRCA mutation carriers, the impact of pregnancy and breastfeeding remains controversial. Women should be encouraged to complete childbearing at early age and discuss thorough breast follow-up during pregnancy and breastfeeding if they decide to maintain their breasts. In BRCA-mutated BC patients, subsequent pregnancy following adequate treatment and follow-up does not seem to increase the risk of BC recurrence and should not be discouraged.*

### Question 2: Does carrying a *BRCA* pathogenic variant impact ovarian reserve and reproductive potential in healthy women?

A major concern among *BRCA1/2* pathogenic variant carriers is the potential higher risk of premature ovarian insufficiency (POI) [22, 23]. Most of the available preclinical evidence suggests that *BRCA* mutations could directly accelerate ovarian aging, reducing the ovarian reserve both quantitatively and qualitatively [24–29]. BRCA1 and 2 are known to be involved in DNA repair mechanism, through ATM-mediated regulation of the DNA double-strand breaks (DSBs) repair [23, 30]. DNA DBS repair mechanisms have a relevant role in ovarian aging; a decrease in their efficiency causes not only an accelerated apoptotic loss of follicles with lethal mutations, but also an increase in meiotic errors and reduced oocyte quality, with an increased number of aneuploidies [23]. There is preclinical evidence that transgenic mice with defective *BRCA* genes have a reduced ovarian response to stimulation and a diminished reproductive potential [30].

In humans, data about fertility in *BRCA* pathogenic variant carriers remain controversial. The majority of the available case-control studies did not report a significant difference in fertility outcomes (i.e., spontaneous abortions and parity) among *BRCA* carriers and non-carriers [31–34]. Fewer studies reported differences in favor of non-carriers [35, 36] with different limitations and confounders (e.g., use/not use of hormonal contraceptives, younger age, and study design). Levels of anti-Müllerian hormone (AMH) are considered a quantitative marker of ovarian reserve, although not predictive of chances of spontaneous pregnancy [37]. In some studies, the levels of AMH were found to be significantly lower in women carrying pathogenic variants in *BRCA1* [24–26, 30] or *BRCA2* [27, 29] or both genes [28], while other studies reported no significant difference with controls [36, 38]. Clinical studies describing a decreased oocyte quality in human carriers of *BRCA* pathogenic variants (i.e., an increase in aneuploidies) are still lacking, while age at natural menopause among *BRCA* carriers is difficult to ascertain, because of various types of selection bias, diverse control groups, and the small population of the studies [39–42]. A further issue related to the shortened window of reproductive opportunity is the recommendation of RRSO at a young age [6]. Although salpingectomy with delayed oophorectomy has been suggested as an option to preserve ovarian function, this strategy is not the gold standard and it should not be recommended [43]. Given these different issues, healthy carriers should be advised not to delay pregnancy beyond 35 years of age [44].

Notably, data about fertility parameters in male carriers of *BRCA* pathogenic variants are very scarce. Spermatogenesis is different from oogenesis. DNA repair mechanisms have the ability to correct DNA alterations only at the germ cell level [45]. Simhadri et al. reported how a mutant PALB2 protein unable to bind BRCA1 in male mice reduced fertility, due to impaired meiosis and increased germ cells apoptosis [46]. However, semen parameters and gonadal function have been never specifically studied in this population.

*Recommendation 2: A potential negative impact of BRCA pathogenic variants on women ovarian reserve and reproductive potential cannot be excluded and should be discussed; however, no strong conclusions can be drawn from existing data. Healthy carriers should be advised to not delay pregnancy beyond 35 years*.

### Question 3: Does carrying a *BRCA* pathogenic variant affect ovarian reserve and reproductive potential in BC patients?

Limited data exist about fertility outcomes in *BRCA*-mutated BC patients [47]. Oktay et al. first described in 2010 a diminished ovarian response and lower number of oocytes for FP in *BRCA*-mutated cancer patients [48]. Since then, a few more studies found a worse quantitative response to controlled ovarian stimulation (COS) in this cohort [48–50], while others reported no difference with not mutated BC patients [51–53]. Similarly, some studies reported lower AMH levels at BC diagnosis in *BRCA*-mutated patients [30, 50, 54], while others did not report a significant difference [52, 53, 55, 56]. A reduced ovarian reserve and a reduced quantitative response to ovarian stimulation would have strong implications for the risk of reduced efficacy of emergency oocytes retrieval and cryopreservation. More research efforts are needed in this field to provide clearer evidence on the need to personalize the oncofertility counseling of women with breast cancer carrying a germline pathogenic variant in *BRCA* genes.

*Recommendation 3: A potential negative impact of BRCA pathogenic variants on women ovarian reserve and reproductive potential including a diminished response to ovarian stimulation in BC patients cannot be excluded and should be discussed during the oncofertility counseling; however, no strong conclusions can be drawn from existing data.*

### Question 4: Are *BRCA*-mutated patients with cancer at higher risk of treatment-induced gonadotoxicity as compared to cancer patients without *BRCA* pathogenic variants?

Young cancer patients requiring cytotoxic drugs are at risk of a negative impact on their ovarian function and reserve that depends on their age at the time of treatment, dosage, and chemotherapy regimen [57]. Due to both the potential high risk of POI in *BRCA*-mutated patients due to a possible impairment of their ovarian reserve even before starting anticancer therapies and the key role of DNA damage-induced follicle death [58], it can be hypothesized that these patients would be particularly sensitive to the gonadotoxic effect of antineoplastic drugs. Facing this critical issue during oncofertility counseling of *BRCA*-mutated patients who are candidates for chemotherapy at a young age is mandatory but very demanding because of the lack of clear evidence. Valentini et al. conducted a survey of 1954 *BRCA1/2*-mutated women who were treated for BC to assess the impact of chemotherapy on the risk of developing treatment-induced POI in this specific subgroup of patients [59]. Chemotherapy-induced POI was defined as ≥ 2 years of amenorrhea commencing within 2 years after initiating chemotherapy. The authors reported a statistically significantly higher proportion of chemotherapy-induced amenorrhea among *BRCA2* pathogenic variant carriers than *BRCA1* ones. Even excluding patients taking tamoxifen (most numerous among the *BRCA2* cohort), the likelihood of chemotherapy-induced amenorrhea remained significantly different compared to patients who did not undergo chemotherapy. Anyway, for neither subgroup, the probability of amenorrhea was higher than that of BC patients without *BRCA* pathogenic variants [59].

A study by Lambertini et al. in patients receiving anthracycline- and cyclophosphamide-based chemotherapy investigating AMH levels up to 3 years after diagnosis showed no additional detrimental effect by the presence of a deleterious germline *BRCA* pathogenic variant on the reduction in AMH levels following cytotoxic therapy [55].

Currently, in *BRCA*-mutated BC patients, platinum agents are often added to standard anthracycline- and taxane-based chemotherapy regimens [60, 61]. Moreover, PARP inhibitors are now available treatment options for *BRCA*-mutated patients with advanced OC and BC [62] and are being studied also in the early setting [63]. Recent data suggest that olaparib could reduce ovarian reserve in mice [64]; therefore, the potential detrimental effect of PARP inhibitors on women ovarian reserve should be further investigated.

*Recommendation 4: The available limited evidence does not demonstrate an increased risk of chemotherapy-induced POI in BRCA carriers but the overall risk remains significant for all patients and fertility preservation should be discussed with all women diagnosed at reproductive age. No data exist on the gonadotoxicity of newer treatment options in these patients.*

### Question 5: Is co-administration of gonadotropin-releasing hormone agonists (GnRHa) during chemotherapy effective for ovarian function preservation in *BRCA*-mutated women with cancer?

Even if young cancer survivors with a *BRCA* pathogenic variant are counseled to undergo RRSO with subsequent iatrogenic POI, the maintenance of ovarian function up to the time of risk-reducing surgery may have a great positive impact on their quality of life, especially in women diagnosed at a very young age. To date, the use of temporary suppression of ovarian function with GnRHa during (neo-)adjuvant chemotherapy is the only recommended medical strategy to preserve ovarian function in young BC patients [65]. The three largest randomized studies on this topic (PROMISE-GIM6, POEMS-SWOG S0230, and OPTION trials) showed similar results with a significant reduction in the risk of developing treatment-induced POI in patients receiving GnRHa during chemotherapy [66–68]. A recent meta-analysis reported that the use of GnRHa during chemotherapy for early BC patients was associated with an absolute 16% reduction in POI rates (from 30.9 to 14.1%; *p* < 0.001) and a higher number of post-treatment pregnancies (37 patients (10.3%) in the GnRHa group vs. 20 patients (5.5%) in the control group; *p* = 0.03) [65]. This effect was observed irrespective of hormone receptor status, patients’ age at the time of diagnosis, type, and duration of chemotherapy [65]. Nonetheless, temporary ovarian suppression with GnRHa during chemotherapy should not be considered an alternative to oocytes/embryos cryopreservation as a strategy for FP (see recommendation 5). Anyway, it may be offered as an additional option following cryopreservation options or when they are not accessible [57, 69]. In patients with cancers other than breast cancer, the use of GnRHa has shown to protect ovarian function in OC patients but not in women with hematological malignancies [70, 71].

It is important to note that there is very limited efficacy data in the specific cohort of patients with cancer and carrying germline *BRCA* pathogenic variants, with only one study reporting the protective effect of GnRHa use during chemotherapy in 4 carriers with BC [69]. There is no biological or clinical rationale to support a different recommendation in patients with cancer carrying or not germline *BRCA* pathogenic variants.

*Recommendation 5: Temporary ovarian suppression obtained by administering GnRHa during (neo-)adjuvant chemotherapy should be offered to all patients with cancer who wish to preserve ovarian function, including BRCA carriers diagnosed years before the recommended age of risk-reducing surgery. Most of the available efficacy data exist in women with BC not carrying BRCA pathogenic variants. GnRHa use during chemotherapy does not replace established fertility preservation methods.*

### Question 6: Is oocyte/embryo cryopreservation safe among young women with a *BRCA1/2* pathogenic variant?

In the setting of both *BRCA*-mutated healthy carriers and patients with cancer, oocyte and embryo cryopreservation should be discussed for different reasons: the first group may benefit from this technique to lengthen the fertile widow (including after the RRSO). The second one may benefit to preserve their reproductive potential before starting a gonadotoxic therapy and/or adjuvant endocrine therapy (ET, which implies a delay in childbearing) [72]. Moreover, it would give all *BRCA* pathogenic variant carriers the possibility to access preimplantation genetic diagnosis for monogenic diseases (PGT-M) [47, 73]. In spite of these critical issues, both the counseling and the use of this FP option are still suboptimal, essentially for safety concerns [74–76].

Three major mechanisms seem to be involved in the carcinogenic effects of estrogens on the breast: stimulation of cellular proliferation through their receptor-mediated hormonal activity, direct genotoxic effects by increasing mutation rates through a cytochrome P450-mediated metabolic activation, and induction of aneuploidy. Since the *BRCA1* and *BRCA2* genes are involved in DNA repair, it could be postulated that the potential effect of estrogens on mammary tissue is aggravated in *BRCA1/2* mutation carriers [77, 78]. To date, there is no evidence that controlled ovarian stimulation (COS) is linked to an increased risk of breast and high-grade OC in the general infertile population [79, 80]. Noteworthy, a recent nationwide cohort study showed a statistically significantly 1.8-fold higher risk of borderline ovarian tumors in women who underwent COS [81]. Perri et al. did not find an increased risk of OC in 164 healthy carriers who underwent fertility treatments versus 909 not treated for infertility [82]. Similarly, Derks-Smeets et al. reported that BC risk was not increased in 76 *BRCA*-pathogenic variant healthy carriers after IVF, compared to controls [77]. While these results are encouraging, they are based on small cohorts and limited follow-up time.

As for BC patients, modified protocols using letrozole in estrogen-sensitive tumors are recommended to reduce estrogen serum concentration by more than 50%, without affecting the number of mature oocytes retrieved or their fertilization capacity and cancer prognosis [57, 83–86]. In a recent survey, 42% of the interviewed BC oncologists were unsure about the safety of COS in *BRCA*-mutated BC patients [7]. However, Kim et al. reported safety outcomes in 120 BC patients versus 217 controls, showing that the 5 years survival was not affected by FP procedures also in the specific cohort of women with a germline pathogenic variant in *BRCA* [86].

*Recommendation 6: Despite the lack of exhaustive data, oocyte and embryo cryopreservation following COS can be considered a safe option that should be proposed both to BRCA-healthy carriers and patients with cancer interested in fertility preservation, whenever feasible. The use of letrozole to suppress supra-physiologic estrogens can be used safely without a reduction in the number or quality of oocytes.*

### Question 7: Is ovarian tissue cryopreservation (OTC) a safe and feasible option in young women with newly diagnosed cancer and carrying a *BRCA* pathogenic variant?

OTC is a widespread FP option, which is already considered standard in several countries [87]. It does not require COS, allowing FP in an urgent setting (e.g., when chemotherapy should be started as soon as possible) [88]. The ovarian cortical tissue is retrieved through laparoscopy, cryopreserved, and then re-transplanted into the ovary enabling the woman to search for a pregnancy spontaneously or through IVF [87, 89]. Literature reports live birth rates around 30–40% in experienced centers [87, 89, 90]. Two live births after OTC and re-transplantation are reported in *BRCA*-mutated early BC patients [52, 91].

Limited data are available about this approach in *BRCA*-mutated patients with cancer. OTC raises important safety concerns on transplanting a tissue in a woman at increased risk of subsequent OC. Then, OTC should not be currently offered to known *BRCA*-pathogenic variant carriers as a first choice for FP. Most of the patients who undergo OTC at the time of cancer diagnosis have no information available yet on their *BRCA* status. However, this information should be known before the transplantation of cryopreserved tissue. In this scenario, the most crucial issue is the choice of the transplantation site to ensure that all ovarian tissue is then removed at the time of RRSO [57].

The possibility of in vitro maturation (IVM) of immature oocytes retrieved during ovarian tissue processing is currently being investigated with some promising preliminary results [92], but it is not yet a clinical reality. Similarly, advancements on a fibrin matrix (the “artificial ovary”) to harvest ovarian tissue outside the body are currently at a translational research stage [93].

*Recommendation 7: OTC should not be in principle recommended for known BRCA pathogenic variant carriers because of the potential OC risk associated with the transplantation of ovarian tissue. However, in selected cases and motivated patients diagnosed several years before the recommended age of RRSO, OTC may be considered with caution when other possibilities are not feasible, but special considerations also for the transplantation procedure are needed. IVM is considered a promising but still experimental strategy in this setting.*

### Question 8: Should PGT-M always be discussed with *BRCA*-pathogenic variant carriers of reproductive age?

In 2003, the Ethics Taskforce of the European Society of Human Reproduction and Embryology (ESHRE) defined PGT-M acceptable for late-onset and multifactorial diseases, including hereditary BC and OC [94]. In 2008, the first live birth following PGT-M for *BRCA1* pathogenic variant carrier was reported by Jasper et al. [95]. Then, Derks-Smeets et al. published one of the largest experiences [96], consisting of 70 couples that underwent PGT for *BRCA* pathogenic variants. However, the reported uptake of the technique is still very low [97–99]. On one hand, this may be a consequence of suboptimal knowledge and referral behavior of the involved professionals [100–102]. Data on women’s uptake of the procedure, when proposed, are also conflictual. Recent surveys were focused on the attitudes about PGT-M among women with *BRCA* pathogenic variants. Most of the respondents declared that PGT should be offered, but less than the half would have used this option for themselves [103–106]. Patients’ attitude to PGT depends on their family and/or personal history of cancer, reproductive history, or both. Several couples expressed that financial access was a major barrier to PGT. Nonetheless, even when IVF/PGT was offered at no cost to *BRCA* pathogenic variant carriers, its uptake was low [106]. Indeed, a previous condition of infertility seemed to be one of the most significant predictors of IVF/PGT use, suggesting that *BRCA* status is secondary to infertility in the decision-making process for PGT-M [107].

The ethical issues are maybe the most crucial. The American Society for Reproductive Medicine (ASRM) may justify PGT-M “when the conditions are serious and when there are no known interventions for the conditions, or the available interventions are either inadequately effective or significantly burdensome” [108]. Carrying a *BRCA* pathogenic variant is not a disease, but a condition that increases the risk of developing different cancers later in life. In addition, *BRCA* genes have an incomplete and variable penetrance and the disease is effectively curable in many cases. Despite these arguments, the anxiety and anguish involved in the need for lifetime preventive testing ethically justify PGT “as a matter of reproductive liberty” [109].

Regarding medical safety, children born after PGT seem to have comparable outcomes in terms of general health and development milestones to those born after IVF only and after natural conception in families carrying risk for a monogenic disease [110]. Recently, an increased incidence of pre-eclampsia was reported in pregnancies after trophectoderm biopsy [111]. The causative link behind the procedure and this finding is, however, still to be confirmed in larger cohorts.

All these elements deeply influence women’s choices and physician attitude to PGT-M, making it very hard to develop clear guidelines. Certainly, a better awareness of PGT-M, with both its indications and limits, has been associated with a greater PGT acceptability also among healthcare providers [112], and it will lead to less moral and ethical reservations, putting couples’ autonomy in the center of the decision-making process.

*Recommendation 8: Carriers of BRCA pathogenic variants interested in avoiding the transmission of their mutation to the offspring have to be informed about the possibility to undergo PGT-M. A thorough and balanced genetic and fertility counseling should be offered to all interested carriers, underlying pros and cons of the procedure.*

## Conclusion

Optimal reproductive counseling in *BRCA*-mutation carriers still represents an unmet challenge, and several misconceptions about the topic persist among health care providers and patients.

The question of a systematic FP approach in *BRCA* carriers, even before the onset of cancer, remains an open issue, and several aspects should be considered in the decision-making process:

1. Age at the time of *BRCA*-mutation disclosure;
2. The potential negative effect of *BRCA* pathogenic variants on women ovarian reserve;
3. The possibility to prevent the transmission of *BRCA* pathogenic variant to offspring with the use of PGT-M;
4. The timing of RRSO, according to *BRCA1* or *BRCA2* pathogenic variants and family history;
5. The need of gonadotoxic anticancer treatments and/or a long-lasting adjuvant ET in *BRCA1/2*-mutation carriers affected by cancer;
6. The safety and feasibility of pregnancy after BC in patients with germline *BRCA* mutations.

During the meeting, with the participation of patient advocacy and delegates from different countries, two main criticisms emerged: (1) patients’ feeling of overload by an excess of information during fertility counseling, resulting in confusion and misunderstandings; (2) a discrepant management also related to the geographical and socioeconomic setting. A well-structured fertility counseling should be proposed to *BRCA1/2* pathogenic variant carriers, both healthy and affected, pre- and post-testing, in order to early identify a personalized and suitable strategy of FP.

On the basis of the discussion, the expert panel has drafted a total of 8 recommendations (Table 1). Although there is a great interest in this field, the lack of large prospective studies on these topics highlights the need of further research efforts. Males who carry *BRCA1/2* pathogenic variants should be included in the counseling process about the risk of transmission and the possible use of PGT-M. Awareness should be implemented, also among medical professionals, regarding all the mentioned reproductive health-specific issues of *BRCA1/2* pathogenic variant carriers, from fertility to PGT to pregnancy after cancer, including indications to contraception and hormonal menopause therapy.

**Table 1:** The 8 recommendations drafted by the expert panel

Recommendations	
1) In healthy *BRCA* mutation carriers, the impact of pregnancy and breastfeeding remains controversial. Women should be encouraged to complete childbearing at early age and discuss thorough breast follow-up during pregnancy and breastfeeding if they decide to maintain their breasts. In *BRCA*-mutated breast cancer patients, subsequent pregnancy following adequate treatment and follow-up does not seem to increase the risk of breast cancer recurrence and should not be discouraged. 2) A potential negative impact of *BRCA* pathogenic variants on women ovarian reserve and reproductive potential cannot be excluded and should be discussed; however, no strong conclusions can be drawn from existing data. Healthy carriers should be advised to not delay pregnancy beyond 35 years. 3) A potential negative impact of *BRCA* pathogenic variants on women ovarian reserve and reproductive potential including a diminished response to ovarian stimulation in breast cancer patients cannot be excluded and should be discussed during the oncofertility counseling; however, no strong conclusions can be drawn from existing data. 4) The available limited evidence does not demonstrate an increased risk of chemotherapy-induced POI in *BRCA* carriers but the overall risk remains significant for all patients and fertility preservation should be discussed with all women diagnosed at reproductive age. No data exist on the gonadotoxicity of newer treatment options in these patients. 5) Temporary ovarian suppression obtained by administering GnRHa during (neo-)adjuvant chemotherapy should be offered to all patients with cancer who wish to preserve ovarian function, including *BRCA* carriers diagnosed years before the recommended age of risk-reducing surgery. Most of the available efficacy data exist in women with breast cancer not carrying *BRCA* pathogenic variants. GnRHa use during chemotherapy does not replace established fertility preservation methods. 6) Despite the lack of exhaustive data, oocyte and embryo cryopreservation following COS can be considered a safe option that should be proposed both to *BRCA*-healthy carriers and patients with cancer interested in fertility preservation, whenever feasible. The use of letrozole to suppress supra-physiologic estrogens can be used safely without a reduction in the number or quality of oocytes. 7) Ovarian tissue cryopreservation should not be in principle recommended for known *BRCA* pathogenic variant carriers because of the potential ovarian cancer risk associated with the transplantation of ovarian tissue. However, in selected cases and motivated patients diagnosed several years before the recommended age of RRSO, ovarian tissue cryopreservation may be considered with caution when other possibilities are not feasible, but special considerations also for the transplantation procedure are needed. IVM is considered a promising but still experimental strategy in this setting. 8) Carriers of *BRCA* pathogenic variants interested in avoiding the transmission of their mutation to the offspring have to be informed about the possibility to undergo PGT-M. A thorough and balanced genetic and fertility counseling should be offered to all interested carriers, underlying pros and cons of the procedure.	

## Data Availability

Not applicable

## Abbreviations

BC: Breast cancer
OC: Ovarian cancer
FP: Fertility preservation
POI: Premature ovarian insufficiency
DSBs: Double strands breaks
AMH: Anti-Müllerian hormone
RRSO: Risk reducing salpingo-oophorectomy
COS: Controlled ovarian stimulation
GnRHa: Gonadotropins releasing hormone analog
ET: Endocrine therapy
PGT-M: Preimplantation genetic diagnosis for monogenic diseases
IVF: In vitro fertilization
OTC: ovarian tissue cryopreservation
IVM: In vitro maturation
ESHRE: European Society for Human Reproduction and Embryology
HBOC: Hereditary breast and ovarian cancers
ASRM: American Society for Reproductive Medicine
